# Effects of Eicosapentaenoic Acid and Docosahexaenoic Acid on Chylomicron and VLDL Synthesis and Secretion in Caco-2 Cells

**DOI:** 10.1155/2014/684325

**Published:** 2014-05-28

**Authors:** Yue Wang, Qiaowei Lin, Peipei Zheng, Lulu Li, Zhengxi Bao, Feiruo Huang

**Affiliations:** Department of Animal Nutrition and Feed Science, College of Animal Science and Technology, Huazhong Agricultural University, Wuhan 430070, China

## Abstract

The present research was undertaken to determine the effects of EPA (20 : 5 n-3) and DHA (22 : 6 n-3) on chylomicron and VLDL synthesis and secretion by Caco-2 cells. Cells were incubated for 12 to 36 h with 400 **μ**M OA, EPA, and DHA; then 36 h was chosen for further study because EPA and DHA decreased de novo triglycerides synthesis in a longer incubation compared with OA  (*P* < 0.01). Neither the uptake nor oxidation was different in response to the respective fatty acids (*P* > 0.05). Compared with OA, intercellular and secreted nascent apolipoprotein B48 and B100 were decreased by EPA and DHA (*P* < 0.01). Both DHA and EPA resulted in a lower secretion of chylomicron and VLDL (*P* < 0.01). In contrast to OA, EPA and DHA were preferentially incorporated into phospholipids instead of triacylglycerols (*P* < 0.01). These discoveries demonstrated that exposure of DHA and EPA reduced the secretion of chylomicron and VLDL partly by regulating the synthesis of TG and apoB.

## 1. Introduction

Hypertriglyceridemia causes or exacerbates a multitude of health problems and has been proved to be a potential risk factor contributing to metabolic syndrome [1, 2]. A significant discovery about marine fish oils abundant in n-3 polyunsaturated fatty acids (n-3PUFAs) is their efficacy to lower plasma triacylglycerol (TG) level [3, 4]. This has engendered considerable interest in the reason for their hypotriglyceridemic effect. It is generally accepted that this well-documented effect is attributed to increased TG clearance from circulation and decreased hepatic triglyceride synthesis [3]. Taking into account that n-3PUFAs have an effect on apolipoprotein B and lipoprotein metabolism in hepatocytes [5–7], it is theoretically possible that n-3PUFAs could also alter lipid metabolism in enterocytes, which contributes to decreasing postprandial triglyceride level.

As one of the TG-rich lipoprotein particles, chylomicron (CM) which is exclusively produced in the enterocytes is responsible for transporting exogenous lipids to the periphery. Once absorbed dietary fatty acids are reconstituted into TG, the TG forms prechylomicron with newly synthesized apolipoprotein B48 (apoB48), followed by further process in the Golgi. Finally chylomicron is transported to the basolateral membrane [8]. The other TG-rich lipoprotein particle is VLDL which is also produced in the enterocytes mostly during fasting [9]. Both of them are the major lipoproteins secreted in the intestine.

Caco-2 cells differentiated on filters are more metabolically active than cells grown on plastic [10]. Taking their multiple biological functions into account, confluent Caco-2 cell monolayers are considered to be an excellent in vitro cell model to study lipoprotein metabolism [11]. The regulation of fatty acids on lipoprotein synthesis and secretion has already been investigated by Caco-2 cells; however, additional work is obviously needed in view of conflicting results. Except for one isolated study demonstrating that incubation with EPA does not alter apoB mass [12], an influx of EPA is reported to result in diminished apoB mass compared with OA [13, 14]. Disparity in the various studies is ascribed to differences in experiment design, including type of fatty acid substrates, concentrations of fatty acids, incubation times, contents of medium (nutritional substrates), stages of differentiation, and culture condition (growth supports). In the present study, we attempted to mimic the in vivo situation by culturing cells on semipermeable membranes, adding physiological concentration of fatty acids and prolonging incubation period.

The influence of eicosapentaenoic acid (EPA) and docosahexaenoic acid (DHA) on lipoprotein synthesis and secretion by Caco-2 was investigated to identify fatty acid-specific alteration in lipoprotein metabolism, which could throw light upon the hypolipidemic effect of EPA, DHA, and related n-3PUFAs. Although this issue has been examined before by incubation with EPA, this is the first time to demonstrate a decrease in secretion and cellular accumulation of both de novo TG and nascent apoB48/B100 by Caco-2 cells in response to DHA. Despite distinctive metabolism pathways, EPA and DHA have been proved to exert similar effect on lipoprotein synthesis and secretion.

## 2. Materials and Methods

### 2.1. Materials

Cell culture media and supplements were obtained from Invitrogen, Carlsbad, CA, USA. 6-well polycarbonate transwell filter inserts with 3 *μ*m pores were purchased from Corning, Corning Inc., NY. [1,2,3-^3^H]glycerol (200 Ci/mol), [l-^14^C]EPA (52 Ci/mol), [1-^14^C]DHA (56 Ci/mL), and [l-^14^C]OA (58 Ci/mol) were obtained from Amersham, Oakville, ON, Canada. Essentially fatty acid-free bovine serum albumin (BSA) was purchased from Sigma Chemical Co., St. Louis, MO. Eicosapentaenoic acid and docosahexaenoic acid (95% pure) were generously provided by Cell Signaling Technology, Inc. (Beverly, MA, USA). TLC plates (Silica gel F 1500) were purchased from Schleicher and Schuell, Dassel, Germany.

### 2.2. Cell Culture

Caco-2 cells were obtained from American Type Culture Collection, Rockville, MD, and cells at passage not more than 38 were maintained in Dulbecco's modified Eagle's medium (4.5 g/L glucose) supplemented with 20% fetal calf serum, 1% glutamine, penicillin (100 IU/mL), streptomycin (100 pg/mL), and 1% nonessential amino acids in a 5% CO_2_ atmosphere at 37°C. Monolayer cells used for subculture were grown in plastic flasks and were split from the flasks using 0.25% trypsin containing 1 mM EDTA when they reached 80%–90% confluence. According to the method mentioned before [15], for differentiation, Caco-2 cells were suspended and seeded at a density of 1.2 × 10^6^ cells/mL on the apical side of transwell filter inserts and the medium was changed every other day for 21 days to allow for differentiation of Caco-2 into enterocytes. In order to evaluate differentiation of monolayers, we measured transepithelial electrical resistance (Millicell-ERS, Millipore Corp., Bedford, MA) and the electrical resistance was approximately 500 Ω/cm^2^, indicating that the cells achieved tight junction formation.

### 2.3. Preparation of Fatty Acids/Albumin Complexes

Complexes of fatty acids combined with bovine serum albumin (BSA) were prepared as described by others [16]. Briefly, excess NaOH was added to the stock solutions of fatty acids dissolved in 95% ethanol to prepare sodium salts of fatty acid. Then ethanol was removed by evaporating under nitrogen, and the sodium fatty acid was dissolved with medium supplemented with required aliquots of BSA. The ratio of fatty acids to albumin was 4 : l. After being stirred for 30 min, the mixtures were sterilized by passage through a 0.22 *μ*m filter and stored at 4°C.

### 2.4. Accumulation and Secretion of Newly Synthesized TG

The method of determining triglyceride synthesis and secretion was described before [14]. Prior to the experimental incubation, cells were washed twice. M199 containing 10 mM AT-2-hydroxyethylpiperazine-JV′-2-ethanesulfonic acid (HEPES), pH 7.4, without serum was placed in the bottom wells, and the DMEM containing 20% delipidated FCS and fatty acid/BSA complexes was added to the top wells. It is noteworthy that control cells received the DMEM containing all components except the fatty acid. To measure the TG synthesis and secretion rate at the times indicated in the figures, four hours before terminating the experiment, [1,2,3-^3^H]glycerol (13 pCi/mL) was added to the apical chamber. After the incubation, the cells were washed and scraped carefully from the filter by a rubber policeman. Lipids in the cells and the basal medium were extracted with chloroform/methanol (2 : 1, vol/vol), and the chloroform extract was washed with methanol/water (1 : 1, vol/vol). The chloroform extract was dried under a stream of nitrogen and applied to silica-gel G plates in 125 *μ*L chloroform. The plates were developed in hexanes/diethyl ether/acetic acid/methanol (170 : 30 : 2 : 2, vol/vol/vol/vol), and the lipids were visualized by exposure to iodine vapor. The bands corresponding to triglycerides were scraped from the plate and counted by liquid scintillation.

### 2.5. Measurement of Lipid Synthesis and Secretion

Lipid synthesis and secretion were assayed as previously described [17, 18]. Briefly, radiolabeled [l-^14^C]OA, [1-^14^C]EPA, or [1-^14^C]DHA was solubilized in fatty acid-free BSA (BSA/fatty acids, 1 : 4 (mol : mol)). The final fatty acid concentration was 400 *μ*M. Cells were first washed with PBS, and the [^14^C] fatty acid-containing medium was added to the upper compartment. At the end of a 36 h incubation period, cells were washed and then scraped with a rubber policeman in a PBS solution containing antiproteases (phenylmethylsulfonyl fluoride, pepstatin, EDTA, aminocaproic acid, chloramphenicol, leupeptin, glutathione, benzamidine, dithiothreitol, sodium azide, and Trasylol, all at a final concentration of 1 mM). An aliquot was taken for lipid extraction by standard methods [19] in the presence of unlabeled carrier (phospholipids (PL), monoglycerides, diglycerides, triglycerides (TG), free fatty acids, free cholesterol (FC), and cholesteryl ester (CE)).

The various lipid classes synthesized from fatty acids were then separated by TLC using the solvent mixture of hexane, ether, and acetic acid (80 : 20 : 3, vol/vol/vol), as previously described [19]. The area corresponding to each lipid was scratched off the TLC plates, and the silica powder was placed in a scintillation vial with Ready Safe counting fluid (Beckman, Fullerton, CA). Radioactivity was then measured by scintillation counting (LS 5000 TD, Beckman). Cell protein was quantified by the Bradford method, and results were expressed as dpm per milligram of cell protein. Lipid secreted in the basolateral compartment was analyzed and quantified, as described above, after centrifugation (2,000 rpm for 30 min at 4°C) to remove cell debris.

### 2.6. Uptake and Oxidation of OA, EPA, and DHA

Measurements of uptake and oxidation of fatty acids were performed as described previously [10]. Cellular uptake and formation of acid-soluble products of [l-^14^C]OA (1 *μ*Ci/mL), [1-^14^C]EPA (1 *μ*Ci/mL), or [1-^14^C]DHA (1 *μ*Ci/mL) were determined. Medium (0.5 mL) and 0.25 mL of cell homogenate were treated with equal volumes of HClO_4_ (1 M). The mixture was centrifuged at 3500 rpm for 10 min and samples from the supernatants were counted. The precipitates were resuspended, washed with 2 mL 1 M HClO_4_, and resolubilized in 1.0 mL of saline containing sodium dodecyl sulfate (SDS) (70 mM) and Triton X-100 (10%). More than 98% of intact fatty acids added were precipitated with this method. A set of no-cell controls was analyzed together with the experiment samples.

The oxidation of fatty acids to acid-soluble products and CO_2_ was measured. Acid-soluble products were mostly ketone bodies, Krebs cycle intermediates, and acetylcarnitine. Medium containing 1.6 mL serum-free DME medium, HEPES (20 mM), [l-^14^C] specific fatty acid (1 *μ*Ci/mL, 400 *μ*M), L-carnitine hydrochloride (0.5 mM), and gentamicin (50 pg/mL) was added to the apical side of Caco-2 cells and incubated for 36 h. The basolateral membrane surface was exposed to 2.5 mL of the incubation media without fatty acids. A special tight plexiglass bell was developed to measure formation of CO_2_ released from Caco-2 cells cultured on filters. The bells had a center well (Kontes, Vineland, NJ) containing 300 *μ*L phenylethylamine-methanol 1 : l (v/v) and a folded filter paper and were closed with stopper tops (Kontes). Four hundred *μ*L perchloric acid (2.8 M) was added to the cell monolayer in the apical well and then incubated at room temperature for 60 min to trap all ^14^CO_2_. At the end of the incubation the well was cut off and counted (liquid scintillation spectrometry). The acid-soluble radioactivity was measured in the HClO_4_ extract. The mixture was centrifuged at 3500 rpm for 10 min and samples of 1 mL of the supernatants were counted. The supernatant excess was neutralized with potassium phosphate buffer (0.5 M, pH 6.5), and ketone bodies (P-hydroxybutyrate and acetoacetate) were measured.

### 2.7. De Novo Apolipoprotein Synthesis

The effect of fatty acids on newly synthesized and secreted ApoB48 and Apo B100 was assessed as described previously [20]. The cells plated on filter membranes and cultured for 21 days were incubated with 400 *μ*M OA, EPA, or DHA. The basal chambers were washed and filled with DMEM without serum or fatty acids. After 35 hours, the apical medium was aspirated and the cells were rinsed in methionine-free M199. They were then incubated for 1 hour in methionine-free M199 that contained the fatty acids attached to albumin. Methionine-free M199 was added to the basal well. One hour later, [^35^S] methionine (100 *μ*Ci/mL) (Amersham Life Sciences, 50 mCi/mmol) was added to the apical well. After being incubated with the label for 30 min, the cells were rinsed in ice-cold PBS containing 10 mM methionine and scraped gently from the filter in 0.2 mL buffer A. The cells were sonicated for 10 seconds and centrifuged for 5 minutes at 13,000 g in a refrigerated microfuge. The medium and cell lysates were supplemented with the antiprotease cocktail. To assay a considerable amount of de novo apolipoprotein synthesis, the material from two wells was pooled.

### 2.8. Immunoprecipitation of Apolipoproteins

The medium and cell lysates were first supplemented with unlabeled methionine to act as a carrier (final concentration, 0.1 mM). Immunoprecipitation was performed in the presence of excess polyclonal antibodies to human apolipoproteins (Boehringer Mannheim) at 4°C overnight [20]. Samples were then washed with Nonidet P-40 (0.05%). They were subsequently centrifuged and resuspended in sample buffer (1.2% SDS, 12% glycerol, 60 mM Tris, pH 7.3, 1.2% b-mercaptoethanol, and 0.003% bromophenol blue) and analyzed by a linear 4–15% polyacrylamide gradient preceded by a 3% stacking gel, as described previously. Radioactive molecular weight standards (Amersham Life Sciences) were run in the same conditions. Gels were sectioned into 2-mm slices and counted after an overnight incubation with 1 mL of Beckman tissue solubilizer (0.5 N quaternary ammonium hydroxide in toluene) and 10 mL of liquid scintillation fluid (Ready Organic, Beckman). Results for each apolipoprotein studied were expressed as percent nmol/mg protein to assess the specific effect of various fatty acids on apolipoprotein synthesis and secretion.

### 2.9. Isolation of Lipoproteins

For the determination of secreted lipoproteins, Caco-2 cells were incubated with the lipid substrate as described above, in the presence or absence of fatty acids. The medium supplemented with antiproteases (as described above) was first mixed with a plasma lipid carrier (4 : 1, vol/vol) to efficiently isolate de novo lipoproteins synthesized. The lipoproteins were then isolated by sequential ultracentrifugation using a TL-100 ultracentrifuge (Beckman), as described previously [19]. Briefly, chylomicrons were isolated after ultracentrifugation (20,000 rpm for 20 min). Very low-density lipoprotein (VLDL) (1.006 g/mL) was separated by spinning at 100,000 g for 2.26 h with a tabletop ultracentrifuge 100.4 rotor at 4°C. Each lipoprotein fraction was exhaustively dialyzed against 0.15 M NaCl and 0.001 M EDTA, pH 7.0, at 4°C for 36 h.

### 2.10. Statistical Analysis

Data were presented as means ± SE. Differences between group means were determined by a one-way ANOVA using the computer program GraphPad Instat (version 2.03; GraphPad Software Inc., San Diego, CA). When significant variations were found, the Tukey-Kramer multiple comparisons test was performed. Differences were considered significant at *P* < 0.05.

## 3. Results

### 3.1. Time Course for the Effect of Fatty Acids on Accumulation and Secretion of Newly Synthesized Triacyl[^3^H]glycerol

**Figure 1:**
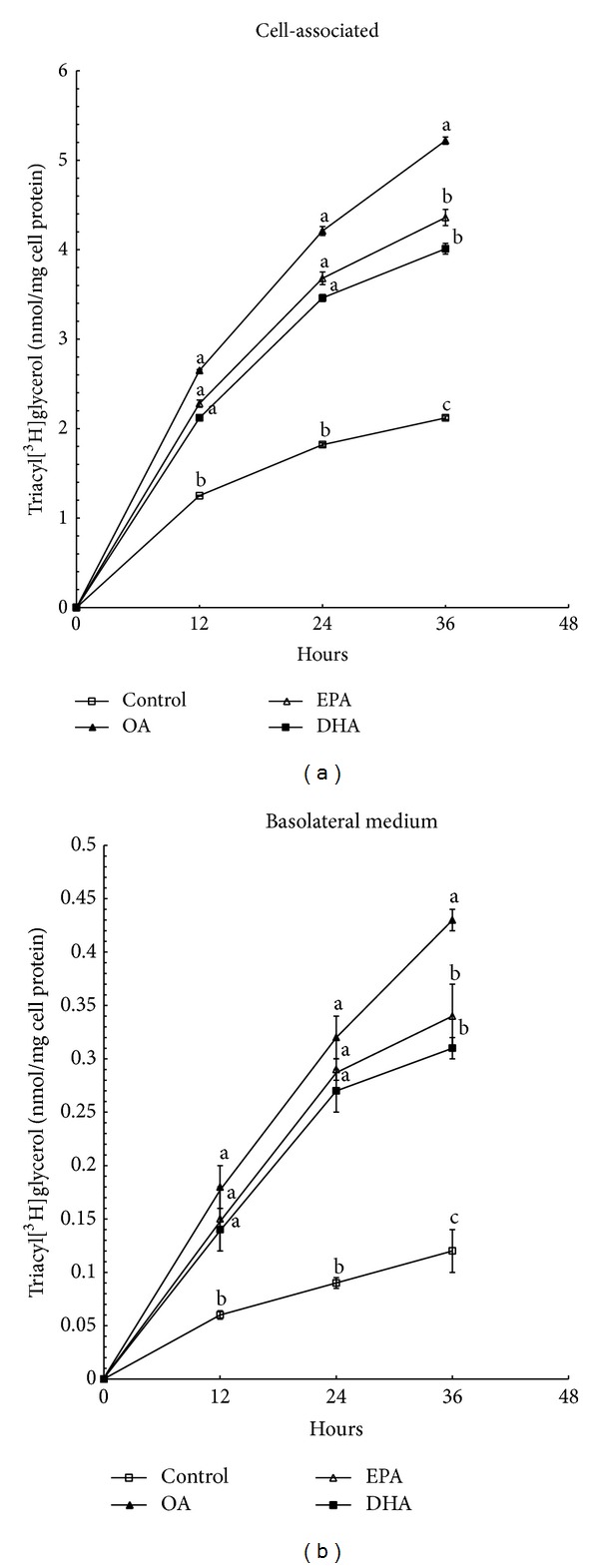
Effect of OA, EPA, and DHA on Caco-2 cell triacyl[^3^H]glycerol accumulation and secretion. The cells plated on filter membranes and cultured for 21 days were incubated with DMEM containing delipidated FCS in the absence or presence of 400 *μ*M OA, EPA, or EHA, respectively, for the time specified in the figure. The incorporation of [1,2,3-^3^H]glycerol into triacyl[^3^H]glycerol accumulated in cells (a) and secreted into basolateral medium (b) was estimated as described in Section 2. OA: oleic acid; EPA: eicosapentaenoic acid; DHA: docosahexaenoic acid. Data are expressed as mean ± SD of 6 different experiments. Means without a common letter differ between fatty acids, *P* < 0.01.

The accumulation and secretion of triacyl[^3^H]glycerol (TG) increased during the whole incubation period and indicated no definite plateau (Figure 1). Compared with the control, 400 *μ*M OA, EPA, and DHA all significantly increased (*P* < 0.01) the accumulation (Figure 1(a)) and secretion (Figure 1(b)) of triacyl[^3^H]glycerol at any time. Remarkably, there was no difference (*P* > 0.05) at early time points (12 h and 24 h) in the presence of 400 *μ*M EPA or DHA, as compared to OA. When the incubation time prolonged to 36 hours, compared with OA, Caco-2 cells incubated with 400 *μ*M EPA significantly caused 16.5% and 21% reductions in the levels of triacyl[^3^H]glycerol synthesis and secretion (*P* < 0.01). Meanwhile, the reduction caused by 400 *μ*M DHA is 23.2% and 28%, respectively (*P* < 0.01). Additionally, there was no difference at any time point between EPA and DHA (*P* > 0.05).

The fatty acid-specific alteration in the synthesis and secretion of TG became pronounced when the incubation time was broadened to 36 h. Thus, the 36 h treatment was chosen for further experiments.

### 3.2. Uptake and Oxidation of OA, EPA, and DHA

Oxidation is another important pathway for fatty acid metabolism within cells. If a particular fatty acid was being diverted to the oxidation pathway and utilized for providing energy, less of it would obviously be available for esterification. Therefore, the uptake and subsequent oxidation of OA, EPA, and DHA were measured in Caco-2 cells.

**Figure 2:**
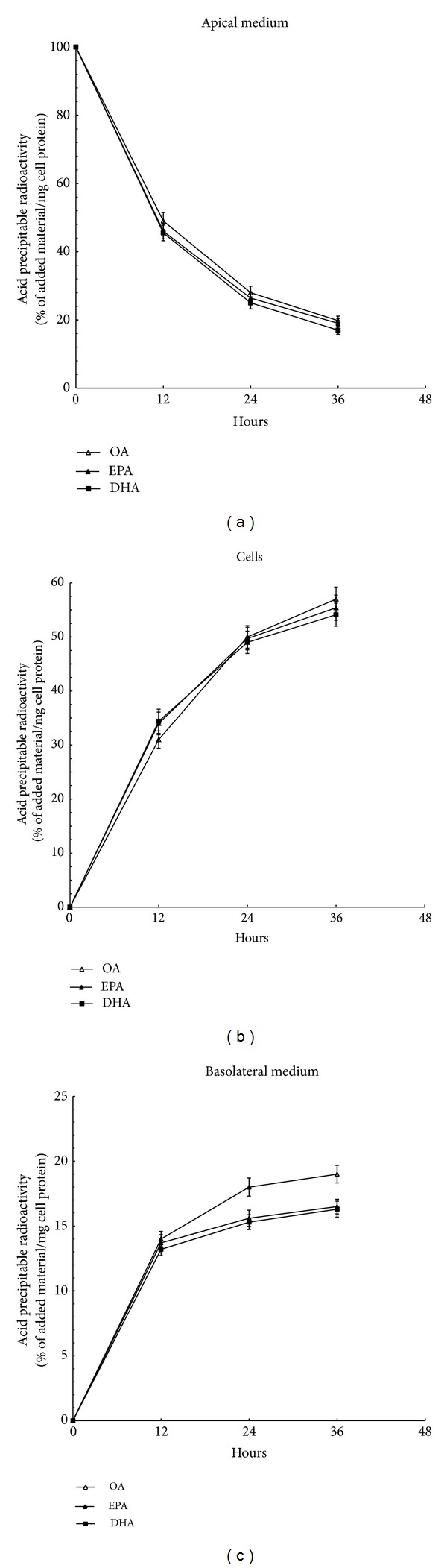
Percent uptake of EPA, DHA, and OA by Caco-2 cells. Caco-2 cells plated on filter membranes and cultured for 21 days were incubated in serum-free DME medium containing 400 *μ*M [l-^14^C]OA, [l-^14^C]EPA, or [l-^14^C]DHA. After 12, 24, and 36 h of incubation, acid-precipitable products were determined from the apical medium (a), cells (b), and basolateral medium (c). The results are expressed as % of added acid-precipitable radioactivity/mg cell protein. Data are expressed as mean ± SD of 6 different experiments. Means without a common letter differ, *P* < 0.01.

400 *μ*M [l-^14^C]OA, [l-^14^C]EPA, or [l-^14^C]DHA was added to Caco-2 cells in the apical chambers. The disappearance of labeled fatty acids from the media (Figure 2(a)) and the cellular uptake (Figure 2(b)) were similar during the incubation period (*P* > 0.05). During the initial 12~36 h approximately 50~80% of the fatty acids had entered the cells. Acid-precipitable products in the basolateral medium reflected the amount of radioactive fatty acids secreted from the Caco-2 cells (Figure 2(c)). The contents of acid-precipitable products in basolateral media were similar in cells incubated with OA, EPA, or DHA (*P* > 0.05).

**Figure 3:**
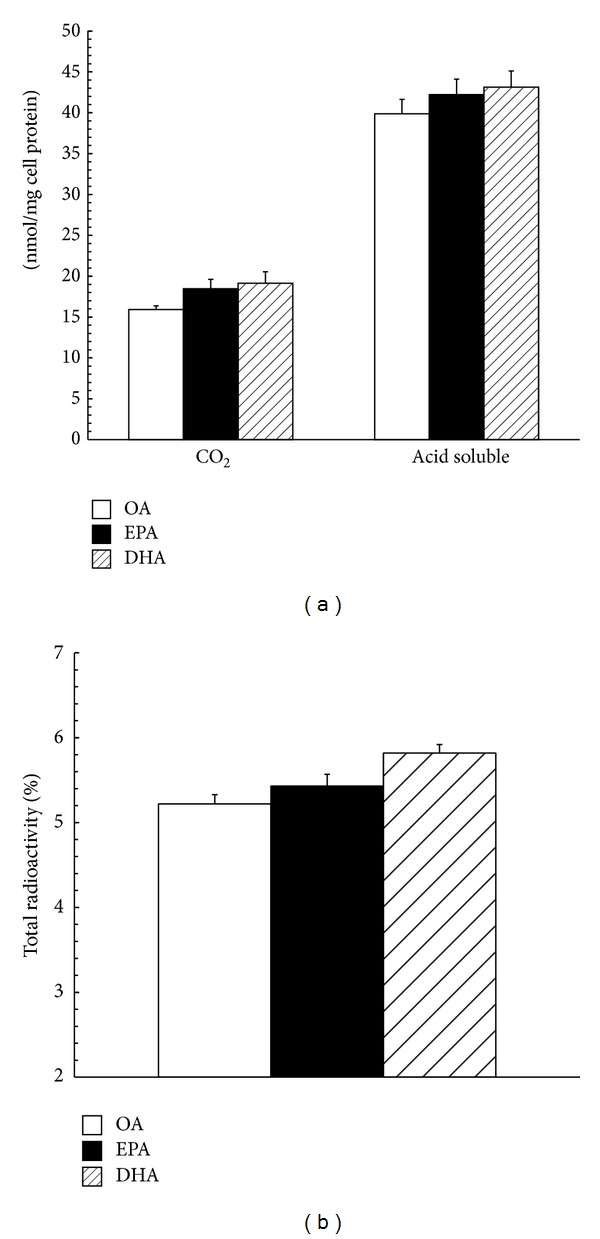
Oxidation of EPA, DHA, and OA in CaCo-2 cells. The cells plated on filter membranes and cultured for 21 days were incubated in serum-free DME medium containing 400 *μ*m [l-^14^C]OA, [l-^14^C]EPA, or [l-^14^C]DHA for 36 h. (a) CO_2_ was collected and acid-soluble radioactivity in the incubation medium was measured as described in Section 2. (b) The sum of CO_2_ and acid-soluble radioactivity as % of total added radioactivity is given. Data are expressed as mean ± SD of 6 different experiments. Means without a common letter differ, *P* < 0.01.

When incubating Caco-2 monolayers for 36 h with 400 *μ*M [l-^14^C]OA, [l-^14^C]DHA, or [l-^14^C]EPA, a similar amount of radioactivity was released as CO_2_ and acid soluble products into the medium (*P* > 0.05), although less than 6% of the lipids were oxidized (Figure 3).

### 3.3. Incorporation of the Fatty Acids into Cellular and Medium Lipids

**Figure 4:**
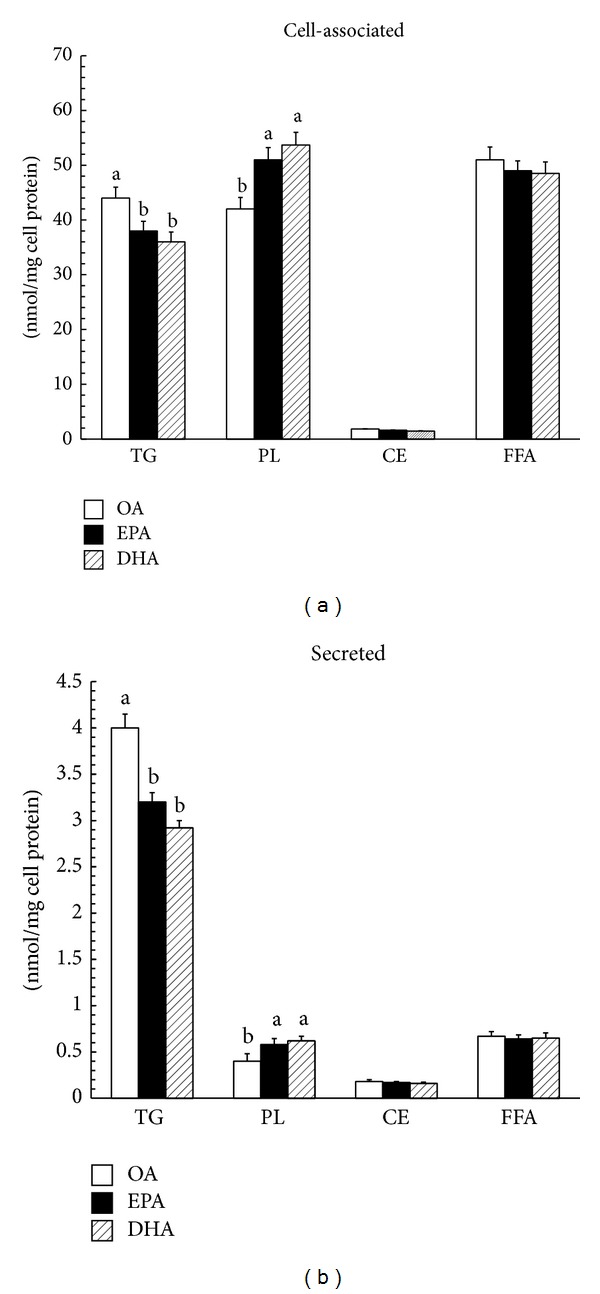
Incorporation of [l-^14^C]OA, [l-^14^C]EPA, and [l-^14^C]DHA into cell-associated and secreted lipids. Caco-2 cells plated on filter membranes and cultured for 21 days were incubated with [l-^14^C]OA, [l-^14^C]EPA, or [l-^14^C]EHA for 36 h. Cells were harvested and basolateral medium was collected for cell-associated (a) and secreted (b) lipids as described in Section 2. OA: oleic acid; EPA: eicosapentaenoic acid; DHA: docosahexaenoic acid; TG: triacylglycerol; PL: phospholipid; FFA: free fatty acids; CE: cholesteryl ester. Data are expressed as mean ± SD of 6 different experiments. Means without a common letter differ, *P* < 0.01.

Because the esterification of a fatty acid into a particular lipid within cells could determine its ultimate fate, the esterification and distribution of the three fatty acids in Caco-2 cells were determined (Figure 4). Determination of [^14^C] fatty acid incorporation into cellular lipids indicated that the major intracellular products were PL and TG, with a low level of CE synthesis. However, TG was the major secretory product in the medium. The percent of the fatty acids which were incorporated into the total esterified lipids, that is, phospholipids, triacylglycerols, and cholesteryl esters, was similar (96%). However, there were significant differences in the distribution of the fatty acids within the cellular lipids. Substantially more OA was incorporated into cellular and medium triacylglycerols compared to EPA (32% versus 27%) or DHA (32% versus 26%) (*P* < 0.01). In contrast, EPA and DHA were preferentially incorporated into phospholipids (37% versus 30%; 38% versus 30%) compared with OA.

### 3.4. Effect of the Fatty Acids on Caco-2 Cell Nascent ApoB48 and ApoB100 Intracellular Accumulation and Secretion

ApoB48 was synthesized exclusively by the small intestine, while apoB100 was synthesized by the liver. In contrast to enterocytes which mainly synthesize apoB48, Caco-2 cells mainly synthesized apoB100 [21]. However, more and more investigations revealed that lipid transport involving CM was a property of intestinal epithelial cells and was not totally dependent on apoB48 expression, since apoB100 could substitute for this function [22, 23]. For these reasons, we paid attention to both apoB48 and apoB100 to investigate the effect of fatty acids on Caco-2 cell apolipoprotein synthesis and secretion.

**Figure 5:**
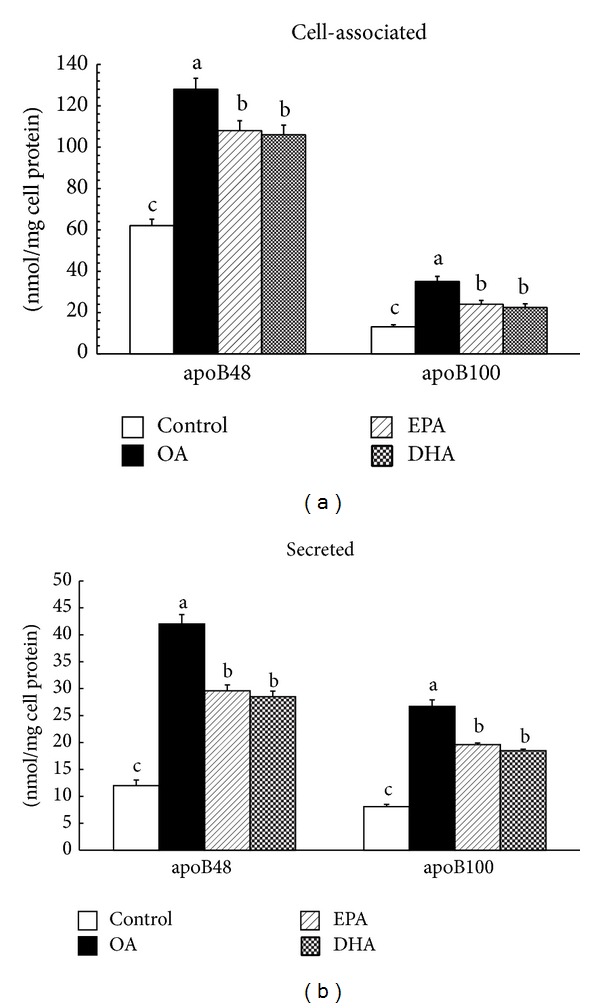
Effect of OA, EPA, and DHA on Caco-2 cell nascent apolipoprotein synthesis and secretion. The cells plated on filter membranes and cultured for 21 days were incubated with 400 *μ*M OA, EPA, or DHA for 35 h. After 35 hours, cells were incubated in methionine-free medium with albumin alone or respective fatty acid for 1 h. Labeled [^35^S] methionine was added to the apical well. Apolipoproteins B-48 and B-100 were analyzed after the incubation with [^35^S] methionine by SDS gel electrophoresis. The cells were harvested and basolateral medium was collected for de novo cell (a) and medium (b) apolipoprotein synthesis as described in experimental procedures. OA: oleic acid; EPA: eicosapentaenoic acid; DHA: docosahexaenoic acid. Data are expressed as mean ± SD of 6 different experiments. Means without a common letter differ between fatty acids, *P* < 0.01.

Although the three fatty acids all increased the incorporation of label into either cellular (Figure 5(a)) or medium (Figure 5(b)) apoB48 and apoB100 compared with controls (*P* < 0.01), less labeled apoB accumulation and secretion by cells incubated with DHA and EPA were observed (*P* < 0.01). Moreover, the incorporation of labeled methionine into cellular and medium apoB48 and B100 was not different between EPA and DHA (*P* > 0.05).

### 3.5. Effect of the Fatty Acids on Caco-2 Cell Lipoprotein Secretion

**Figure 6:**
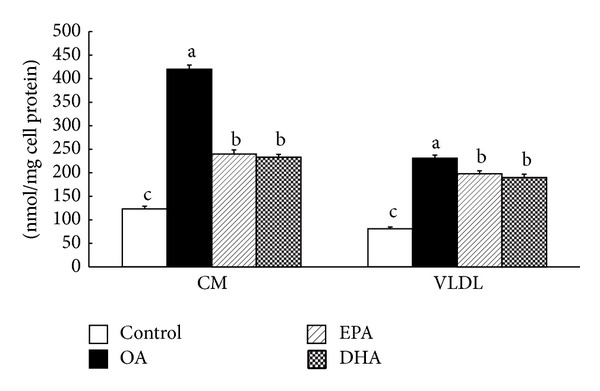
Effect of OA, EPA, and DHA on Caco-2 cell lipoprotein synthesis and secretion. Caco-2 cells plated on filter membranes and cultured for 21 days were incubated with OA, EPA, and DHA for 36 h. Cells were harvested and basolateral medium was collected for lipoprotein isolation as described in Section 2. CM: chylomicrons; VLDL: very low-density lipoprotein; OA: oleic acid; EPA: eicosapentaenoic acid; DHA: docosahexaenoic acid. Data are expressed as mean ± SD of 6 different experiments. Means without a common letter differ between fatty acids, *P* < 0.01.

Control cells were incubated with 100 *μ*M albumin without the fatty acid. Compared with the control, the fatty acids all increased CM and VLDL secretion (Figure 6; *P* < 0.01). However, CM was significantly induced in fatty acids-treated cells in contrast to VLDL which is stimulated to an extent not so distinct to control. Remarkably, compared with OA, 400 *μ*M EPA or DHA at 36 h, respectively, resulted in a lower secretion of chylomicrons and VLDL (*P* < 0.01). Additionally, there was no significant difference between EPA and DHA (*P* > 0.05).

## 4. Discussion

As EPA and DHA are major n-3PUFAs found in fish oil, and OA is the most potent stimulator of newly synthesized TG [24], they are chosen for our experiments. The results of synthesis and secretion of labeled TG, as determined by [1,2,3-^3^H]glycerol incorporation, increase in a time-dependent manner without a plateau. It is noteworthy that in a short-term incubation, the synthesis and secretion of labeled TG mass are similar in cells treated with the three fatty acids. It is not until incubation times up to 36 h that the inhibition of TG synthesis and secretion by DHA and EPA is observed. In a previous report, investigators also find that, after 24 h of incubation, synthesis and secretion of labeled TG by Caco-2 cells incubated with either EPA or OA are similar [10, 25]. Additionally, in cultured rat hepatocytes which are used to study the effect of n-3PUFAs on TG synthesis associated with VLDL in liver, n-3PUFAs stimulate hepatic TG synthesis to an extent similar to OA when hepatocytes are incubated for 2 h [13]. It is likely that a longer incubation time is important to distinguish the effects of specific fatty acid on TG synthesis. Studies based on healthy people demonstrate that chronic (but not acute) intake of fish oil may inhibit the synthesis or secretion of CM from the gut, further confirming the in vitro observations. On the other hand, it is confirmed that DHA reduces hepatic synthesis and secretion of TG by decreasing the activity of acyl-coenzyme A: 1,2-diacylglycerol acyltransferase involved in TG synthesis [26]. Moreover, EPA and DHA can limit the availability of diacylglycerol for esterification by inhibiting hepatic phosphatidate phosphohydrolase activities [27]. Thus, we suggest that there is a lag period between the time of incubation of fatty acids and the time required to inhibit TG synthesis. Taking into consideration that fatty acids serve as the precursors to signaling molecules such as steroids and prostaglandins, we speculate that their metabolites, not themselves, play a crucial role in regulation of lipid metabolism. Generally speaking, there is a certain disagreement concerning the effect of molecular chain-length degree of unsaturation on labeled TG synthesis and secretion. Major source of variability in response most likely stems from the variations of cell models, fatty acid concentrations, incubation periods, and culture conditions, especially culture supporters. As discussed before [28], culture conditions significantly affect Caco-2 cells polarity and differentiation, resulting in altering their functions. Other researchers also investigate whether the marked inhibition of new TG synthesis by n-3PUFAs would result in reduction of total TG secretion, and the answer is yes. Total TG mass secreted by cells incubated with n-3PUFA was diminished than that observed in OA-treated cells [14]. Moreover, despite a continuous increase in TG secretion, intracellular TG mass is still significantly accumulated. That is to say, there is a limit as to the amount of TG secreted as lipoproteins.

Our data indicated no marked difference in uptake of fatty acids observed among EPA-treated, DHA-treated, and OA-treated cells, which is in accordance with previous studies in vitro and in vivo [10, 29]. So we rule out the possibility that n-3PUFAs cause reduced TG synthesis and secretion by blocking fatty acids absorption.

Animal experiments show that consumption of fish oil can increase peroxisomal *β*-oxidation [15], whereas our results demonstrate that the three fatty acids are oxidized to a similar extent in Caco-2 cells and the faction for oxidization is only approximately 6%. There is a report drawing the similar conclusion, which demonstrates that in response to EPA and OA only approximately 5% of the lipids are diverted into oxidization pathway and no alteration has been observed between different fatty acid treatments [10]. In fact, both of the experiments are mimicking an acute exposure to fat and not comparable with the studies in a long-term period. When the incubation time with n-3PUFAs prolongs to an extensive period, it is possible that stimulatory effect on fatty acids oxidization will be observed. Our suggestion is supported by the observation that the expression of genes involved in fatty acid oxidation and oxygen consumption rate are increased in DHA-treated Caco-2 cells [15]. Shorter periods of incubation were thought to be too short to detect observable difference. Increased fatty acid oxidation is considered as another potential mechanism to explain decreased substrates for TG synthesis. Whatever, in present studies, the possibility that low rate of TG secretion is due to enhanced oxidation can also be excluded.

Although the suggestion that n-3PUFAs inhibit apoB secretion by several cell models has been made previously, this is the first study that focuses on newly synthesized apoB48 and B100 accumulation and secretion in response to EPA and DHA by Caco-2 cells. Although Caco-2 cells secrete practically no apoB48 when they are grown on plastic, in our hands, a large proportion of apoB48 are synthesized when they are cultured on the filters. In the present study, we reveal that the synthesis and secretion of both labeled ApoB48 and ApoB100 are stimulated by cells in response to the presence of fatty acids. It makes good sense that acute fatty acids influx stimulates apoB synthesis paralleled by similar changes in the apoB secretion, since more absorbed fatty acid must be packed into lipoproteins and then transported into lymph efficiently. Compared with control cells, the secretion and intracellular accumulation of newly synthesized apoB are significantly increased by OA. There is a report that partly agrees with our results, demonstrating an increase in the secretion of newly synthesized apoB100 in HepG2 and Caco-2 cells incubated with OA for 24 h [30]. These observations are inconsistent with previous findings, where the rate of synthesis and secretion of newly synthesized apoB in cells exposed to OA for 18 h or 48 h is similar to control cells [13, 14]. It seems that, in response to an influx of OA, increased rate of apoB synthesis is not necessary since intracellular pool of preformed apoB can be called on to transport TG. However, it is likely that the reason for this disparity is attributed to difference between hepatocytes and intestinal absorptive cells. Moreover, 18 h time point seems too short to observe changes, whereas compared with OA inhibitory effect on labeled apoB48 and B100 synthesis and secretion exerted by EPA and DHA is significant, which is partially substantiated by the observation that EPA causes a significant decrease in the rate of methionine incorporation into newly synthesized apoB within cells and that found in the basolateral medium [14]. Except for our research, no such studies have been performed in Caco-2 cells by using DHA. Numerous observations exist for demonstrating no effect [12] or a decrease [13, 14] in apoB mass secreted by n-3PUFAs, compared with OA. Moreover, apoB mRNA abundance is significantly decreased in cells exposed to n-3PUFAs [14]. These data well support our discovery that the stimulation of fatty acids on apoB synthesis is attenuated by n-3PUFAs; it seems that less apoB is destined for translocation and secretion. The question as to why n-3PUFAs decrease apoB synthetic rates remains to be answered. However, in previous studies, it is reported that the amount of apoB mass within cells is not altered by EPA or OA when the incubation time prolongs to 48 h [14, 30]. It is possible that none of these fatty acids alter the levels of apoB mass within cells despite having stimulated apoB synthetic rate. With the influx of fatty acids, apoB synthesis is stimulated but increased apoB secretion still results in progressive labeled apoB accumulation and constant total apoB mass. Given that the rate of protein synthesis seems to be rate-limiting step for the assembly and secretion of lipoprotein particles, it is possible that the mechanism for the inhibitory effect of n-3PUFAs on plasma triacylglycerol level via reduced apoB synthesis resulted in reduced chylomicron and VLDL assembly. It should also be noted that the ratio of labeled apoB48 to apoB100 accumulated in EPA- and DHA-treated cells was similar to control, suggesting that EPA and DHA do not modify apoB editing. However, Caco-2 cells provided with OA referentially promote accumulation of cellular ApoB100, resulting in a less proportion of apoB48 relative to apoB100. Additionally, it is possible that there is a shift of apoB from the more dense lipoproteins to TG-rich lipoproteins that are responsible for transporting the TG [24].

Our data reaffirm previous findings of other investigators, suggesting that DHA and EPA are preferentially incorporated into phospholipids (PL), in contrast to OA which is mainly integrated into TG. This can partly account for substantially less synthesized TG with EPA and DHA than with OA. The difference in incorporation of the fatty acids into certain lipid classes has been described by Caco-2 cells before [10]. EPA is more potent that OA in inducing TG synthesis, whereas EPA is a poorer substrate than OA for PL synthesis. It is likely that the effect caused by specific fatty acid can be a result of a direct metabolic effect. Maybe the substrate affinity of specific fatty acid for different enzymes involved in TG and PL synthesis can help illuminating our confusion, but no more information is available. Observations based on HepG2 cells disagree with conclusions drawn by Caco-2 cells, arguing that at 4 h incorporation of radiolabeled EPA into cellular TG was greater than the incorporation obtained with radiolabeled OA [5].

As clear from Figure 5, it seems that CM and VLDL productions all increase in the cells exposed to fatty acids. It is obvious that the marked elevation in TG synthesis is concurrent with increase in apoB synthesis rate. The production of VLDL is less sensitive to specific fatty acid addition, since fatty acids all induce VLDL secretion to a similar degree. It is clear that inhibitory effect is exerted by EPA and DHA primarily on CM, but not VLDL.

As the first potential regulatory factor in lipoprotein assembly and secretion, TG synthesis can be a prerequisite to yield enough substrates available for lipoprotein assembly. Apolipoprotein synthesis is an additional critical factor associated with lipoprotein assembly. In this study, we concluded that EPA and DHA inhibit CM and VLDL assembly and secretion in intestinal epithelial cells partly via reducing TG and apoB synthesis, which alleviated hyperlipidemia by directly reducing absorbed TG secretion from enterocytes.
